# Systemic clearance of *p16^INK4a^*‐positive senescent cells mitigates age‐associated intervertebral disc degeneration

**DOI:** 10.1111/acel.12927

**Published:** 2019-03-21

**Authors:** Prashanti Patil, Qing Dong, Dong Wang, Jianhui Chang, Christopher Wiley, Marco Demaria, Joon Lee, James Kang, Laura J. Niedernhofer, Paul D. Robbins, Gwendolyn Sowa, Judith Campisi, Daohong Zhou, Nam Vo

**Affiliations:** ^1^ Department of Orthopedic Surgery University of Pittsburgh Pittsburgh Pennsylvania; ^2^ Department of Pathology University of Pittsburgh Pittsburgh Pennsylvania; ^3^ Department of Pharmaceutical Sciences University of Arkansas for Medical Sciences Little Rock Arkansas; ^4^ Buck Institute for Research on Aging Novato California; ^5^ Department of Orthopaedic Surgery, Brigham and Women's Hospital Harvard Medical School Boston Massachusetts; ^6^ Department of Biochemistry, Molecular Biology and Biophysics, The Institute on the Biology of Aging and Metabolism University of Minnesota Minneapolis Minnesota; ^7^ Department of Physical Medicine and Rehabilitation University of Pittsburgh Pittsburgh Pennsylvania; ^8^ Life Sciences Division Lawrence Berkeley National Laboratory Berkeley California; ^9^ Department of Pharmacodynamics University of Florida Gainesville Florida

**Keywords:** aggrecanolysis, aging, cellular senescence, intervertebral disc, *p16^Ink4a^*, proteoglycan

## Abstract

**Rationale:**

Age‐related changes in the intervertebral discs are the predominant contributors to back pain, a common physical and functional impairment experienced by older persons. Cellular senescence, a process wherein cells undergo growth arrest and chronically secrete numerous inflammatory molecules and proteases, has been reported to cause decline in the health and function of multiple tissues with age. Although senescent cells have been reported to increase in intervertebral degeneration (IDD), it is not known whether they are causative in age‐related IDD.

**Objective:**

The study aimed to elucidate whether a causal relationship exists between cellular senescence and age‐related IDD.

**Methods and Results:**

To examine the impact of senescent cells on age‐associated IDD, we used p16‐3MR transgenic mice, which enables the selective removal of *p16^Ink4a^*‐positive senescent cells by the drug ganciclovir. Disc cellularity, aggrecan content and fragmentation alongside expression of inflammatory cytokine (IL‐6) and matrix proteases (ADAMTS4 and MMP13) in discs of p16‐3MR mice treated with GCV and untreated controls were assessed. In aged mice, reducing the per cent of senescent cells decreased disc aggrecan proteolytic degradation and increased overall proteoglycan matrix content along with improved histological disc features. Additionally, reduction of senescent cells lowered the levels of MMP13, which is purported to promote disc degenerative changes during aging.

**Conclusions:**

The findings of this study suggest that systemic reduction in the number of senescent cells ameliorates multiple age‐associated changes within the disc tissue. Cellular senescence could therefore serve as a therapeutic target to restore the health of disc tissue that deteriorates with age.

## 1 INTRODUCTION

Low back pain (LBP) is a major chronic disorder that significantly reduces the quality of life of the elderly. In the United States, direct and indirect costs associated with LBP, including lost wages and productivity and legal and insurance costs, amount to $100 billion annually (Chiodo et al., n.d.; Sambamoorthi, Tan, & Deb, 2015). Though the exact cause of LBP is not known, the degenerative changes that accompany the intervertebral disc with age are most frequently associated with LBP (Wong, Karppinen, & Samartzis, 2017). The debilitating back pain stemming from intervertebral disc degeneration (IDD) causes loss of mobility and increases the risk of mortality in the elderly (Hirvensalo, Rantanen, & Heikkinen, 2000). Furthermore, disc degeneration is associated with clinical conditions such as herniated discs, spinal stenosis and spondylolisthesis, which are major diagnoses which often require surgical treatment. Therefore, there is great need to understand how aging affects the disc in order to maintain mobility and fitness in the fast‐growing population of elderly.

During aging, the intervertebral disc (IVD) undergoes structural, biochemical and biomechanical changes that ultimately compromise its function. The loss of aggrecan, a major disc matrix proteoglycan (PG), results in reduced capacity to resist compressive forces, a well‐established hallmark of IDD. The disc transforms into a fibrous tissue with age because of increased fragmentation and loss of disc PG, with a concomitant decrease in water content, leading to fissures and decreased disc height (Buckwalter, 1995; Sztrolovics, Alini, Roughley, & Mort, 1997; Vo et al., 2016). Evidence also exists for elevated levels of matrix fragments generated by the proteolytic action of matrix metalloproteinases (MMPs), as well as a disintegrin and metalloproteinase with thrombospondin motifs (ADAMTS), in aged discs (Haidong, Mei, Xu, & Gang, 2014). Expression of major pro‐inflammatory cytokines such as IL‐1ß, IL‐6, IL‐8 and TNF‐α, known to induce gene expression of ADAMTS and MMPs in IVD cells, is also increased in older adult discs when compared to younger or adolescent discs. Additionally, IL‐6 levels are found to be higher in patients with low back pain prompted by disc degeneration (Weber et al., 2016). At the cellular level, cellular senescence has been reported to increase with age in discs (Zhao, Wang, Jiang, & Dai, 2007). Cellular senescence may play a major role in age‐related IDD as the senescent phenotype occurs at the right time and has the right characteristics to drive age‐related disc degeneration.

Cellular senescence is a response to stress and certain physiological signals by which cells cease to divide and adopt a distinct phenotype. This phenotype includes marked chromatin and secretome changes, and increased expression of tumour suppressor genes such as the *p16^Ink4a^* cell‐cycle inhibitor (Campisi, 2005; Melk, Schmidt, Takeuchi, Sawitzki, & Halloran, 2004). The senescence response is a potent tumour‐suppressive mechanism and has also been implicated in organismal aging. Senescent cells accumulate with age in most, if not all, tissues of humans, primates and rodents (Deursen, 2014; Herbig, 2006; Wang et al., 2009). Furthermore, senescent cells are found at higher levels in diseased tissue compared to unaffected tissue in patients with age‐related disorders such as osteoarthritis and type 2 diabetes (Naylor, Baker, & Deursen, 2012). Senescent cells are postulated to promote aging via their senescence‐associated secretory phenotype (SASP); the increased and chronic secretion of a suite of inflammatory cytokines, chemokines and proteases (Coppé, Desprez, Krtolica, & Campisi, 2010), which can disrupt tissue function and structure. Recent reports showing amelioration of several age‐associated pathologies upon senescent cell clearance in naturally aged and transgenic mice strongly support the idea that senescent cells can drive tissue aging (Baker et al., 2011; Baker et al., 2016). The role of cellular senescence in disc aging and degeneration is less firmly established. Published reports showed an increased number of senescent cells with age and degeneration in human discs (Gruber, Ingram, Davis, & Hanley, 2009; Maitre, Freemont, & Hoyland, 2007). However, these correlative studies do not clarify whether senescent cells play a causal role in age‐related disc degeneration.

To test the hypothesis that senescent cells play a causal role in age‐associated disc degeneration, we used the p16‐3MR transgenic mouse model in which *p16^Ink4a^*‐positive senescent cells express the herpes simplex virus thymidine kinase and thus can be selectively eliminated upon treatment with ganciclovir (GCV) (Chang et al., 2016; Demaria et al., 2014). Here, we show that year‐long clearance of senescent cells mitigates age‐associated increases in disc protease, PG matrix fragmentation and PG loss. These results provide compelling evidence that senescent cells are responsible for generating inflammatory cytokines and catabolic proteases that are known to increase with aging in disc, and thus support a causal relationship between cellular senescence and age‐associated disc degeneration.

## 2 RESULTS

### 2.1 Increased cellular senescence and matrix homeostatic imbalance in discs of naturally aging mice

**Figure 1:**
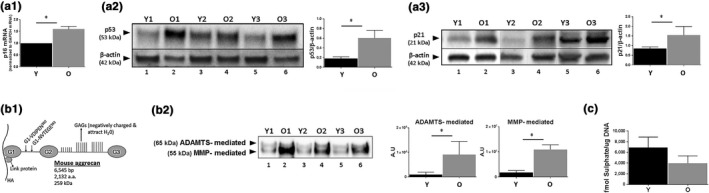
Degenerative changes in intervertebral discs of naturally aged mice. (a) Expression of selected senescent markers, *p16^Ink4a^* (a1), p53 (a2) and p21 (a3) in disc tissue from young (6 month) and old (22 month) mice was determined by Western blotting and qRT–PCR. Graphs on the right of the Western images are quantification of results (a2 and a3) whereby the volume of respective protein band divided by volume of β‐actin band. (b) Increased disc aggrecan proteolysis with age. (b1) A schematic of the mouse aggrecan core protein covalently linked to the sulphate‐rich glycosaminoglycan (GAG) and noncovalently bound to a hyaluronic acid (HA) chain via the link protein. The cleavage sites between G1 and G2 interglobular domains by ADAMTS (G1‐NVTEGE^392^) and MMP (G1VDIPEN^360^) proteases are indicated. (b2) Western blot analysis of aggrecan fragments generated by ADAMTS and MMP proteases with graphs showing quantification results. Representative Western blots for three young and three old mice were shown (panels a2, a3, b2). (c) Proteoglycan synthesis as measured by ^35^S‐sulphate incorporation using whole disc organ cultures. Student's *t* test was used for parametric data (graphs a1, a2, b2), and the Mann–Whitney test was used for two‐variable nonparametric data (graphs a3, c). Data are means ± *SD* of four independent experiments (4 mice) for graphs a1, a3, c and three mice for graphs a2, b2. *p < 0.05. Y = young and O = old. Representative Western images of three different young (lanes 1, 3, 5) and old (lanes 2, 4, 6) mice are shown in panels a2, a3, and b2)

Evidence of increased disc cellular senescence with age in humans, rats and in an accelerated aging mouse model was previously reported. However, it was not known whether cellular senescence also occurs in the discs of naturally aged mice. In order to study the effects of clearance of senescent cells on age‐related IDD in natural aging mice, it is important to determine whether there is increased disc cellular senescence in these animals. Hence, we assessed expression of three key senescence markers, p53, p21 and *p16^Ink4a^*, in the discs of young (6 months) and old (22 months) C57BL/6 mice by Western blot analysis and real‐time quantitative RT–PCR. These cell‐cycle protein regulators promote cell growth arrest and senescence in response to stress. Discs from older mice expressed significantly higher levels of p53 (3×) and p21 (1.5×) protein compared to those from younger mice (Figure 1a2,a3). Due to the lack of specific antibodies for immunodetection of mouse *p16^Ink4a^*, we measured disc p16^Ink4a ^mRNA by real‐time quantitative RT–PCR and found a 1.5× increase in old mice compared to young ones (Figure 1a1). All three markers of cellular senescence, *p16^Ink4a^*, p21 and p53, were significantly elevated in discs of natural aged mice.

A key marker of age‐associated IDD is matrix homeostatic imbalance, particularly enhanced extracellular matrix catabolism. This marker of IDD, while well‐documented in discs of other aging animals and humans, is not well‐documented in natural aged mice. Here, we showed that ADAMTS‐mediated fragmentation and MMP‐mediated fragmentation of aggrecan in older mouse disc were also 10× higher in discs from older, compared to younger, mice (Figure 1b). In contrast, matrix PG synthesis capacity, assessed by ^35^S‐sulphate incorporation using disc organotypic culture, was lower in discs from older, compared to younger, mice (Figure 1c). These in vivo correlations between disc cellular senescence and perturbed matrix homeostasis are consistent with our recent cell culture study demonstrating a matrix imbalance phenotype of senescent disc cells (Ngo, Patil et al., 2017).

### 2.2 Clearance of disc senescent cells using p16‐3MR transgenic mice

**Figure 2:**
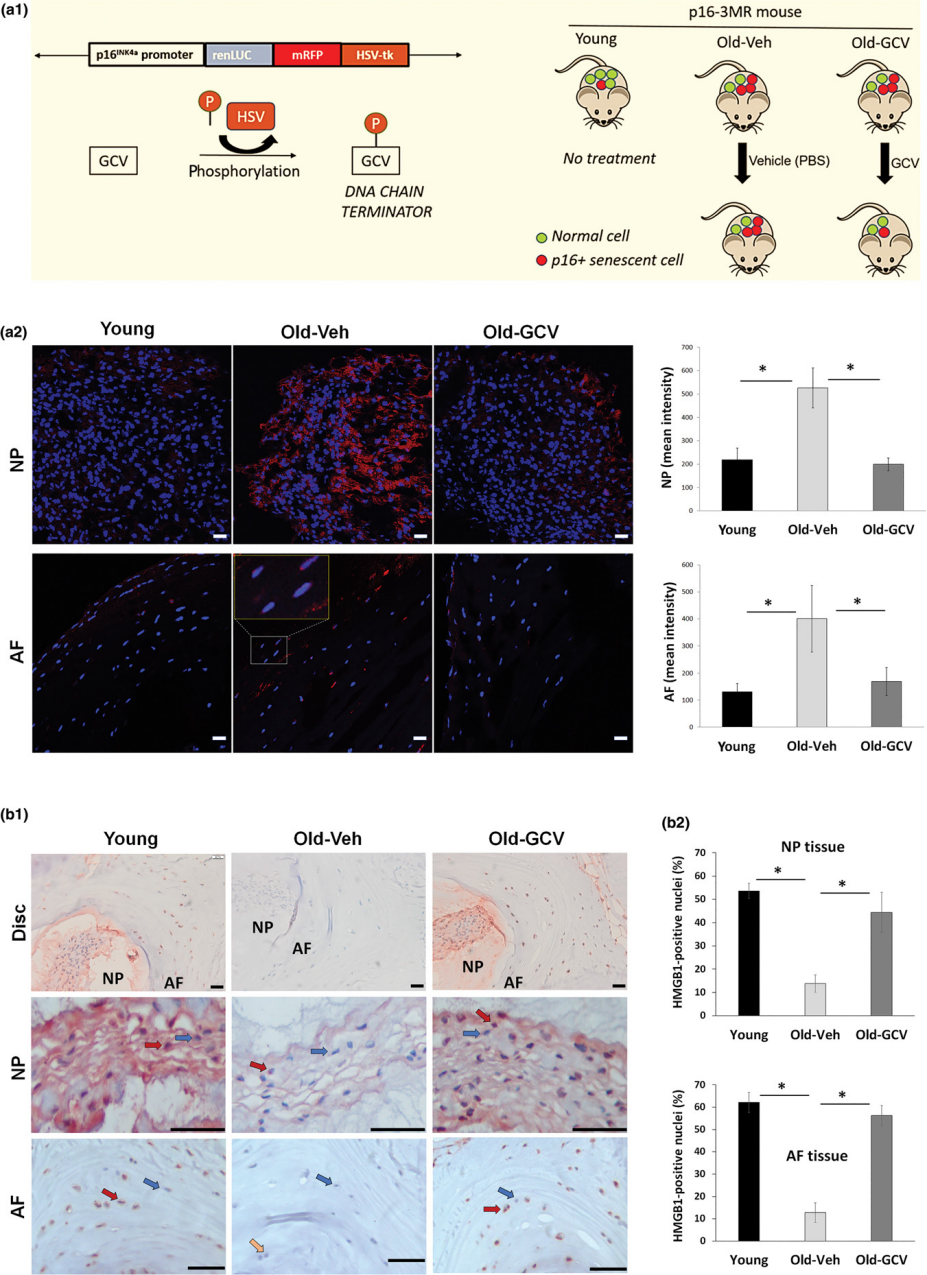
Clearance of senescent cells in p16‐3MR mice. (a1) Schematic of the p16‐3MR transgene and strategy used to selectively kill senescent cells. p16‐3MR mice were sacrificed at 12 months of age (Young) or were administered PBS (Old‐Veh) or GCV (Old‐GCV) for an additional 12 months before sacrifice. Confirmation of elimination of *p16^Ink4a^*‐positive cells in Old‐GCV mice was assessed by examining levels of RFP fluorescence (panel a2) and nuclear HMGB1 protein by IHC (panel b) in AF and NP tissues. Graphs on the right are quantification of the imaging results. Panel b2 shows quantitation of nuclear HMGB1 in disc cells. Data shown are means ± *SEM* of 4 independent experiments (4 mice), *p < 0.05. Examples of cells stained negative (blue arrows), partially positive (yellow arrow) or positive (red arrows) for nuclear HMGB1 are indicated. Scale bars = 10 µm (panel a2) and 50 µm (panel b1)

To test the concept of a causative role for cellular senescence in driving age‐related IDD, we used p16‐3MR transgenic mice (Figure 2a1). These mice contain a transgene encoding a fusion protein consisting of Renilla luciferase (LUC), monomeric red fluorescent protein (mRFP) and herpes simplex virus 1 thymidine kinase (HSV‐TK) collectively driven by the senescent sensitive p16^Ink4a ^promoter. In this p16‐3MR mouse model, *p16^Ink4a^*‐positive senescent cells can be selectively killed by ganciclovir (GCV), a nucleoside analog with a high affinity for HSV‐TK that converts it into a toxic DNA chain terminator, causing fragmentation of genomic and mitochondrial DNA and apoptosis selectively in HSV‐TK expressing senescent cells (Chang et al., 2016; Demaria et al., 2014).

To determine whether our year‐long systemic treatment of p16‐3MR mice with GCV reduces the percent of senescent cells from disc tissue, we measured RFP as a surrogate marker for p16 since it is driven by the *p16^Ink4a^* promoter. In our study, expression of RFP in nucleus pulposus (NP) and annulus fibrosus (AF) tissues of old mice treated with GCV was three times lower compared to old mice treated with PBS, suggesting that GCV reduced the number of *p16^Ink4a^*‐positive senescent disc cells in the old GCV‐treated group (Figure 2a2, Supporting Information Figure S1). Notably, disc RFP expression was higher in old mice treated with PBS than young mice, confirming the increased disc senescence observed in aged WT mice (Figure 2a2).

To further confirm elimination of disc senescent cells by GCV treatment, we measured the level of nuclear HMGB1 staining by immunohistochemistry as loss of nuclear HMGB1 expression is another key marker of cellular senescence (Davalos et al., 2013). Our data showed that most NP and AF cells (~60%) stained positive for nuclear HMGB1 in young mice while only 10%–15% stained in old mice treated with PBS vehicle. In contrast, NP and AF disc tissue of the GCV‐treated mice contained mostly nuclear HMGB1‐positive cells (Figure 2b and Supporting Information Figure S2). These results demonstrate increased disc cellular senescence with age and that the year‐long treatment of p16‐3MR mice with GCV effectively eliminated most of disc senescent cells. However, it is important to note that our systemic GCV treatment eliminates *p16^Ink4a^*‐positive cells in all tissues, not just the disc.

### 2.3 Elimination of senescent cells by GCV treatment improved disc histological features and suppressed disc matrix PG loss in aged mice

**Figure 3:**
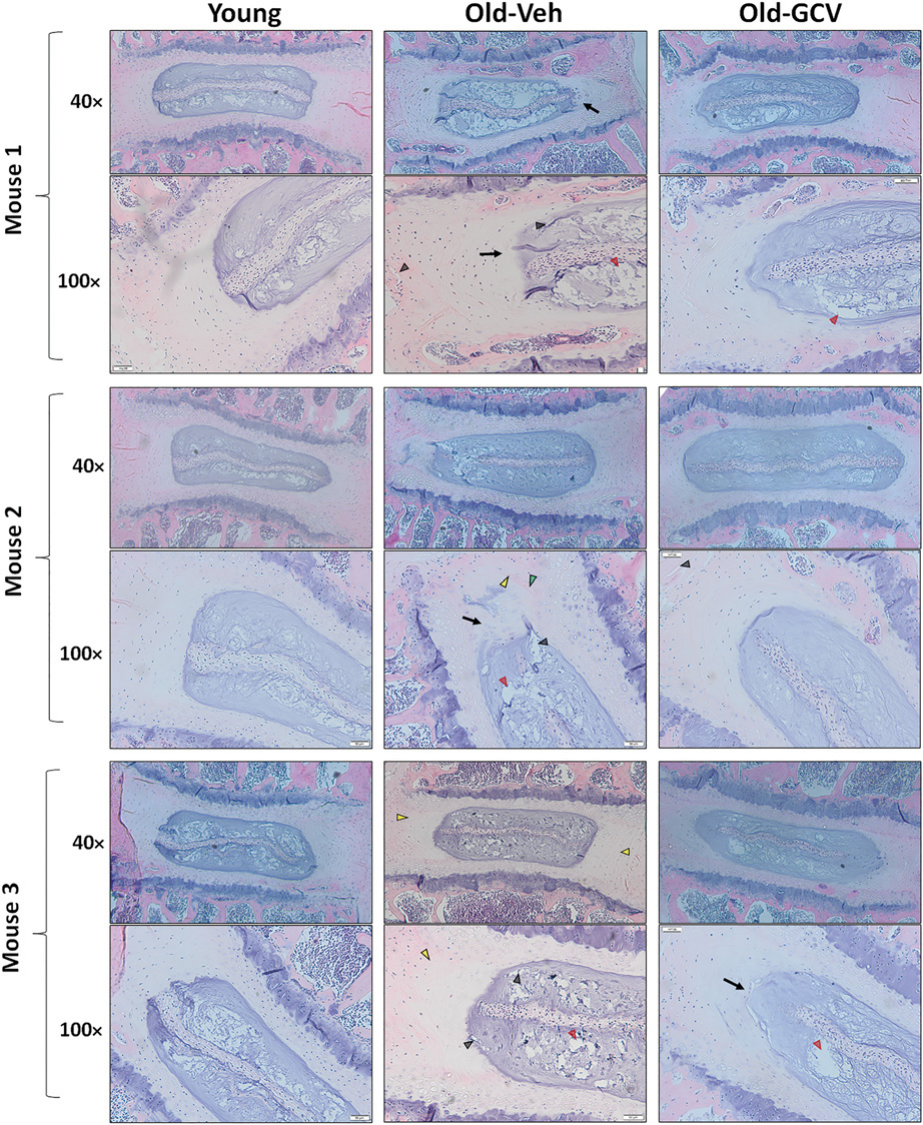
Impact of GCV treatment on gross morphology. H&E staining of lumbar disc was performed to assess the gross morphological changes with aging and GCV treatment. Compared to discs of young mice, discs of old p16‐3MR mice treated with PBS exhibited increasing loss of distinct NP/AF boundary (black arrows), loss of AF structure in which the AF lamellae become less concentric and more serpentine with each lamellae spaced farther apart (yellow arrowheads), loss of NP matrix indicated by large empty space gaps (red arrowheads) and fissures/clefts (black arrowheads). These degradative changes were blunted in the old p16‐3MR mice treated with GCV. Disc sections from three representative mice of each group (p16‐3MR Young, Old‐Veh and Old‐GCV) are shown. Scale bar = 50 μm of H&E‐stained disc sections

To determine whether GCV treatment blunts disc degenerative changes in aged mice, we performed H&E histological analysis to compare the gross morphological disc features between young and old mice. We quantified the changes in several key disc histological features using the histological grading system previously established by Tam and her coworkers for mouse discs (Alvarez‐Garcia, Matsuzaki, Olmer, Masuda, & Lotz, 2017; Tam et al., 2018). Histology grading was done in a blinded manner by three scorers on multiple H&E‐stained disc sections from each of the four individual mice in the Young, Old‐Veh and Old‐GCV group. Compared to young mice, vehicle‐treated old mice exhibited several key degenerative histological changes in their discs, including clefts/fissures, loss of NP cellularity, loss of well‐defined AF‐NP boundary, thinning and loss of NP matrix, and structural disorganization of AF lamellae. Most of these degenerative changes were reduced in GCV‐treated mice (Figure 3), as reflected in the combined histological scores (Table 1).

**Table 1:** Disc histological scores

Score (mean ± *SEM*)	Young	Old‐Veh	Old‐GCV
NP cellularity loss	0.53 ± 0.12	1.11 ± 0.56	0.53 ± 0.35
NP clefts/fissures	1.58 ± 0.30	1.94 ± 0.14	1.33 ± 0.41
AF/NP boundary	0.83 ± 0.42	1.22 ± 0.24	1.13 ± 0.44
AF structure	1.44 ± 0.29	2.28 ± 0.11	1.40 ± 0.31
AF clefts/fissures	0.83 ± 0.44	2.22 ± 0.64	1.60 ± 0.53
Composite score	5.22 ± 0.51	8.78 ± 1.33	6.01 ± 1.22

The improvement in these disc histological features, especially the NP matrix density, by GCV treatment was consistent with the overall increased safranin O staining of NP tissue of GCV‐treated old mice compared to vehicle‐treated mice (Supporting Information Figure S3). The beneficial effect of GCV treatment on disc matrix is also supported by our disc aggrecan mRNA and protein expression data. The GCV‐treated old mice had 37% of the NP aggrecan content of younger mice, whereas vehicle‐treated mice only had 10% of the NP aggrecan content of young mice (Figure 5b). Moreover, improved aggrecan gene expression upon GCV treatment was also observed (Figure 5a).

### 2.4 Clearance of cellular senescence blunted disc matrix PG proteolytic destruction

**Figure 4:**
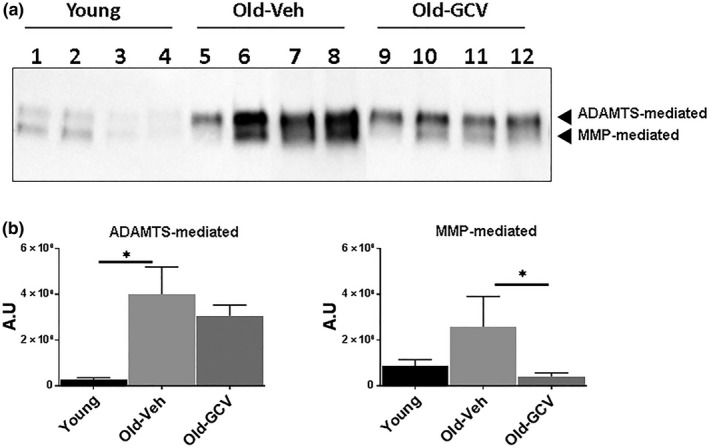
Effects of GCV treatment on disc aggrecanolysis of p16‐3MR mice. (a) Immunoblot of MMP‐ and ADAMTS‐mediated cleavage of aggrecan of discs of young mice (lanes 1–4), vehicle‐treated old mice (lanes 5–8) and GCV‐treated old mice (lanes 9–12). Graphs on right are quantification of aggrecan fragments shown in panel on left. Data shown are mean ± *SD* of 4 independent experiments, *p < 0.05

Disc matrix PG loss is largely due to destruction of aggrecan, the major PG structural constituent of the disc extracellular matrix. Disc aggrecan fragmentation catalysed by MMP and ADAMTS classes of proteases was suppressed in old GCV‐treated mice compared to old vehicle‐treated mice. In fact, MMP‐mediated fragmentation in Old‐GCV mice was comparable to that of young and was six times lower than in Old‐Veh mice. In contrast, the decrease in ADAMTS‐mediated fragmentation of aggrecan in Old‐GCV mice is less dramatic compared to Old‐Veh mice (Figure 4).

**Figure 5:**
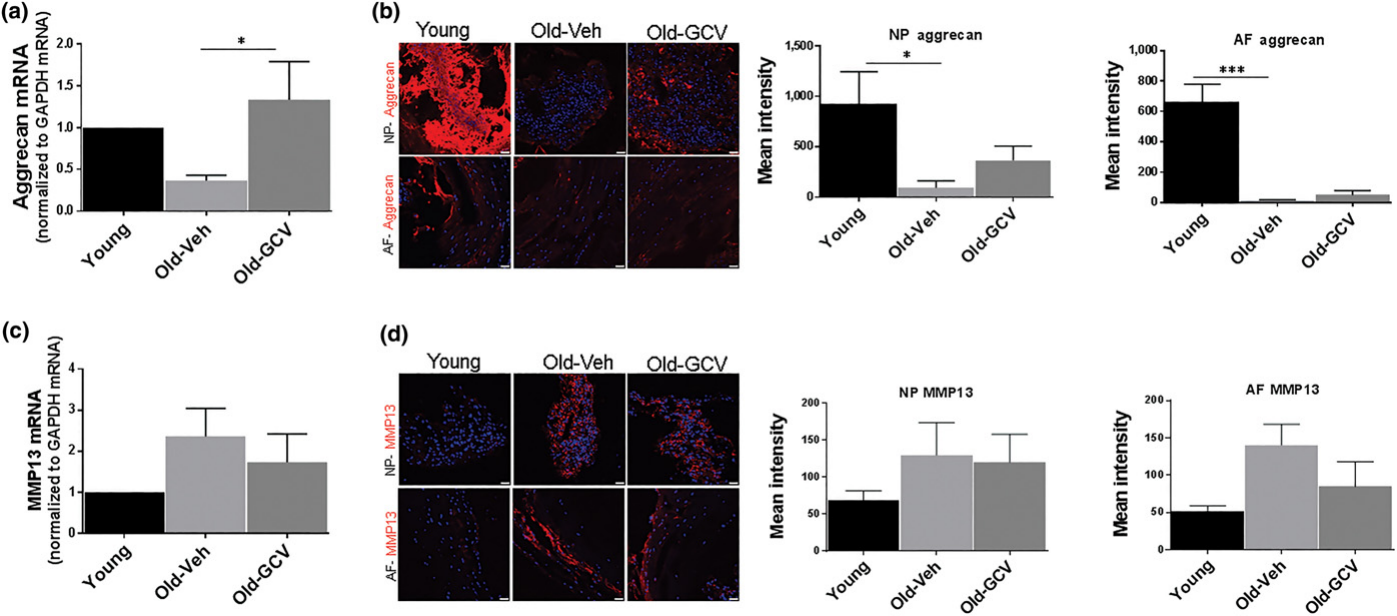
Effects of GCV treatment on aggrecan and MMP13 mRNA and protein levels in intervertebral discs of p16‐3MR mice. Aggrecan mRNA levels were quantified by qRT–PCR (a), and protein was quantified by immunofluorescence signals (b) in nucleus pulposus and annulus fibrosus section of disc tissue. MMP13 expression of whole disc mRNA by qRT–PCR (c) and protein by immunofluorescence (d) in inner nucleus pulposus and outer annulus fibrosus section of disc tissue. Graphs on the right are quantification of the immunofluorescence results. Data shown are mean ± *SEM* of four independent experiments (4 mice), *p < 0.05, ***p < 0.001. Scale bar = 10 μm

One of the major MMPs implicated in disc PG proteolytic destruction and IDD is MMP13 (Roughley, 2004). Disc MMP13 expression, at both the protein and mRNA level, increased with age in old vehicle‐treated mice, but this increase was attenuated by GCV treatment (Figure 5c,d). Hence, the levels of MMP13 expression correlate with the levels of MMP‐mediated aggrecanolysis in disc tissue, implicating MMP13 as the culprit protease responsible for age‐related disc aggrecan degradation. On the other hand, ADAMTS4 is a major aggrecanase responsible for ADAMTS‐mediated proteolysis of aggrecan. We found that disc ADAMTS4 expression was not affected by GCV treatment (data not shown), and hence, this might explain the modest reduction of ADAMTS‐mediated disc aggrecanolysis in GCV‐treated mice (Figure 4). It should be noted that reduced aggrecan fragmentation was also seen in p16‐3MR mice treated with GCV for 6 months to clear senescent cells late in life (Supporting Information Figure S4). However, the effects were not as pronounced, suggesting that prolong senescence clearance is needed to produce beneficial effects.

## 3 DISCUSSION

Cellular senescence and the SASP negatively impact tissue health and contribute to a myriad of age‐associated disorders. Previous studies documented elevated cellular senescence in degenerative discs and a positive correlation between disc cellular senescence level and chronological age in humans (Gruber et al., 2009; Maitre et al., 2007). It was not clear, however, whether cellular senescence was a driver or bystander in disc aging and degeneration in these reports.

Since aging is the major contributor to disc degeneration, we hypothesized that cellular senescence is causal in age‐related disc degeneration. We found elevated levels of senescence markers, together with repressed PG synthesis and increased aggrecan fragmentation, in intervertebral discs of aged mice. Notably, the selective removal of senescent cells ameliorated these degenerative changes in conjunction with suppression of matrix protease expression. These findings suggest that the removal of senescent cells attenuated the age‐associated disc degenerative changes. This is the first study to evaluate and report a direct adverse impact of cellular senescence on intervertebral disc with age.

The increased fragmentation and loss of proteoglycan, specifically aggrecan, are hallmarks of disc degeneration. Matrix degradative changes invariably lead to altered biomechanics and pathologic outcomes such as spine stiffness, spinal stenosis and disabling chronic back pain. Multiple reports show that inflammatory cytokines such as IL‐1ß, IL‐6 and TNF‐α initiate the cascade that culminates in disc degeneration. These cytokines suppress the synthesis of matrix proteins and upregulate production of matrix proteases that include ADAMTS‐4/5, MMP1, MMP2, MMP3, MMP13 and MMP14 (Risbud & Shapiro, 2014). Indeed, in this study, we saw a concomitant reduction in levels of the major disc protease, MMP13, and aggrecan fragmentation in Old‐GCV compared to Old‐Veh mice, suggesting cellular senescence phenotype as the source of the inflammatory proteins that trigger the degeneration cascade. This claim is substantiated by the report published by Ngo et al., which showed decreased PG synthesis capacity, enhanced aggrecan fragmentation, as well as increased production of several cytokines and matrix proteases in oxidative stress‐induced senescent human NP cells (Ngo, Patil et al., 2017). Together, these studies support the causal role for senescent cells in driving matrix homeostatic imbalance in aging discs.

Compared to young p16‐3MR mice, both MMP‐ and ADAMTS‐mediated proteolysis of aggrecan were elevated in aged p16‐3MR mice. However, only MMP‐mediated aggrecanolysis, not ADAMTS‐mediated aggrecanolysis, was significantly reduced in discs of GCV‐treated old p16‐3MR mice. This result is consistent with the effects of GCV on expression of the two predominant matrix proteases in disc tissue, MMP13 and ADAMTS4. MMP13 expression was reduced, while ADAMTS4 remained unaffected. Although the levels of MMP13 were reduced in old p16‐3MR mice treated with GCV, a substantial decrease in levels of IL‐6, a cytokine known to induce MMP (Supporting Information Figure S5; Studer, Vo, Sowa, Ondeck, & Kang, 2011), was not observed. This failure could be due to the fact that IL‐6 and ADAMTS4 are not SASP factors of *p16^Ink4a^*‐positive disc cells and hence are not reduced by GCV administration. Overall, the reduced aggrecan fragmentation seen in GCV‐treated old p16‐3MR mice could be due to elimination of *p16^Ink4a^*‐positive disc cells that overexpress primarily MMPs and not ADAMTS.

To reduce the levels of other catabolic factors that are not decreased upon elimination of *p16^Ink4a^*‐positive senescent cells, it will be important to identify *p16^Ink4a^*‐independent stressor(s) that induce senescence in the disc. Studies published to date suggest that oxidative stress, genotoxic stress and chronic activation of NF‐κB signalling play an important role in propagating age‐related disc degenerative changes (Nasto et al., 2012; Nasto, Robinson et al., 2013; Nasto, Wang et al., 2013). For example, lowering oxidative stress can increase PG content (Risbud & Shapiro 2014). Likewise, inhibition of classical NF‐kB signaling ameliorated several disc aging features, including PG content and cellularity. However, much like these past reported studies, our current study relied on models wherein the stressors and therapeutic treatments had systemic effects, so it is unclear whether these effects occur if the stress or therapeutic intervention is induced only locally in the intervertebral disc tissue. Further investigation is needed to resolve the ambiguity about the relative contributions of systemic vs. local factors to age‐associated disc degeneration.

The observed improvement in disc pathology in p16‐3MR mice treated with GCV is consistent with previous results using treatment of aged mice intermittently with senolytic agents. For example, the senolytic combination of dasatinib and quercetin was shown to not only reduce senescence and improve multiple age‐related pathologies, but also to improve PG content in the disc of progerioid *Ercc1* ^−/∆^ mice (Zhu et al., 2015). Similarly, treatment of *Ercc1* ^−/∆^ mice with the senolytic HSP90 inhibitor 17‐DMAG resulted in not only an extension of healthspan, but also improved PG content (Fuhrmann‐Stroissnigg et al., 2017). Furthermore, intra‐articular injection of the senolytic drug Navitoclax, a Bcl‐2 family inhibitor, was recently shown to slow the progression of age‐associated and post‐traumatic osteoarthritis in aged mice (Jeon et al., 2017). However, the effect of these senolytics on disc pathology in naturally aged mice is unknown.

It is important to note that although we have demonstrated a reduction in the percent of senescent cells and in expression of senescent markers in the disc, we have not demonstrated that there is actually induction of senescent cell death in the disc. The p16‐3MR mice were treated with GCV over a year period, preventing the accumulation of senescent cells in multiple tissues including the disc. Thus, it is possible that the GCV treatment prevented the accumulation of senescent cells in the disc through a cell nonautonomous mechanism, either systemically or locally; that is, GCV‐mediated removal of senescent cells eliminated their paracrine effects of SASP on neighbouring cells (Demaria, Desprez, Campisi, & Velarde, 2015; Robbins, 2017). However, our results unambiguously demonstrate the deleterious impact of senescent cells on intervertebral disc degeneration with natural aging. Notably, we found that *p16^Ink4a^*‐positive senescent cells promote the production of catabolic factors that are known to contribute to age‐associated disc degeneration. As such, our findings indicate that therapeutic approaches involving repopulation with functional IVD cells, growth factor supplementation or gene therapy may face challenges unless the inflammatory milieu created by senescent cells is eliminated from the degenerative disc (Miyamoto et al., 2010; Moriguchi et al., 2016; Nishida et al., 1999; Walsh, Bradford, & Lotz, 2004). In addition, these results suggest that a therapeutic strategy utilizing senotherapeutic agents such as D + Q, 17‐DMAG or Navitoclax could be developed to ameliorate age‐associated disc degeneration (Zhu et al., 2015).

## 4 METHODS

### 4.1 Mice

Naturally aged 6‐month‐old (Young) and 20‐month‐old (Old) C57BL/6 wild‐type mice were obtained from the National Institute of Aging and maintained under specific pathogen‐free conditions at University of Pittsburgh's animal facility. All animal work was approved and done in accordance with University of Pittsburgh's IACUC. Tissues from the p16‐3MR mice were obtained from Dr. Daohong Zhou's group at University of Arkansas for Medical Sciences. Briefly, the p16‐3MR mice were either sacrificed at 12 months (Young) or were treated with either PBS (Old‐Veh) or GCV (Old‐GCV) for additional 12 months (25 mg kg^−1^ day^−1^ for 5 days/cycle, every other 2 weeks) before sacrifice (Studer et al., 2011). Spines and tails isolated from the p16‐3MR mice were immediately frozen using liquid nitrogen and stored at −80°C until shipment.

### 4.2 Quantification of matrix proteoglycan synthesis

Ex vivo culture of functional spine units (FSUs), consisting of vertebrae‐disc‐vertebrae, was established as described (Nasto, Wang et al., 2013). FSUs were cultured in complete medium [F‐12/D‐MEM containing 10% foetal bovine serum, 1% PS (10,000 units/ml penicillin, 10 mg/ml streptomycin) and 25 μg/ml l‐ascorbic acid] for 2 days to equilibrate after the trauma of surgical dissection, followed by a 12‐hr incubation with ^35^S‐sulphate (20 μCi/ml). Proteoglycan synthesis was calculated as fmoles of sulphate incorporated per microgram DNA by as described (Nasto, Wang et al., 2013). Average values from three mice, each analysed in duplicate, were calculated and reported ±1 *SE*.

### 4.3 Histological stain

Isolated lumbar spine segments were decalcified and embedded in paraffin (*Tissue Tek *processor and *Leica *embedder). Seven‐micrometre sections were stained with haematoxylin and eosin (H&E) and safranin O and fast green dyes (Fisher Scientific) by standard procedures and photographed under 40–100× magnification (Nikon Eclipse Ts100). Changes in histological features were evaluated by three blinded scorers using the histological grading system established for mouse discs (Alvarez‐Garcia et al., 2017; Tam et al., 2018). Four mice from each of the three groups, young, old treated with vehicle and old treated with GCV, were analysed; three lumbar sagittal sections from each mouse were scored for the following specific features in the NP and AF as specified by Tam and coworkers.

These include NP cellularity, AF structure, NP matrix structure, distinct AF/NP boundary and fissure/cleft. Normal young disc is defined to have (a) high NP cellularity which is centralized as a single mass of vacuolated cells enclosed within a layer of matrix, (b) normal AF that is aligned in concentric lamellae without disruption to the structure, (c) NP matrix structure in which the matrix mass is continuous and full without significant empty space and (d) well‐defined AF/NP boundary whereby the AF and NP compartments were always distinct. Each feature was scored individually with a score of 0–14; whereby, 0 represented the young health state and 14 represented the maximum degenerative state. A composite score was also calculated by adding all the individual scores (Table 1).

### 4.4 Immunoblotting

Ten tail discs from each mouse were used to assess aggrecan fragmentation utilizing a previously established method using anti‐aggrecan primary antibody (Cat. No. ab36861, Abcam) and anti‐rabbit HRP secondary antibody (Cat. No. 31460, Thermo Fisher; Ngo, Pohl et al., 2017). Expression of p53 protein was examined by extracting protein from spine discs using T‐PER Tissue Protein Extraction Reagent with proteinase inhibitor cocktail as per the manufacturer's instructions (Cat. No 78510, Thermo Fisher) and running a Western blot using the p53 (Cat. No. 2524, *CST*) and β‐actin (Cat. No. PA1‐183, Thermo Fisher) primary antibody and anti‐rabbit HRP secondary antibody (Cat. No. 31460, Thermo Fisher).

### 4.5 Immunofluorescence

Mouse lumbar intervertebral disc tissue was isolated from spines and fixed overnight at 4°C in 2% paraformaldehyde. For immunofluorescent staining, the tissues were cryoprotected with 30% sucrose in PBS overnight at 4°C and then embedded in OCT (Tissue‐Tek). Serial axial plane cryosections were cut at thicknesses of 5 µm. The tissue sections were rehydrated in PBS, permeabilized and blocked with 0.25% Triton X‐100, 10% goat serum and 1% BSA in PBS for 30 min at room temperature. Incubation with primary antibodies (anti‐Aggrecan, Cat. No. abB1031, Abcam; anti‐MMP13, Cat. No. ab39012, Abcam; anti‐IL‐6, Cat. No. PAI‐26811, Thermo Fisher; anti‐RFP, Cat. No. ab62341, Abcam) were carried out overnight at 4°C following blocking. The sections were then incubated with secondary antibodies (Cy3‐conjugated goat anti‐rabbit IgG, Cat. No. 111‐165‐003, Jackson ImmunoResearch Laboratories) for 60 min at room temperature, according to the manufacturer's protocols. Immunostained sections were imaged and analysed using a Nikon instrument A1 confocal laser microscope and NIS‐Elements Microscope Imaging software.

### 4.6 HMGB1 immunohistochemistry

Paraffin‐embedded sections of the lumbar disc we6re used to probe for HMGB1 using anti‐HMGB1 antibodies (1:200 dilution, Cat. No. ab18256; Abcam) and the secondary antibodies (1:500 dilution of biotin‐conjugated goat anti‐rabbit, Cat. No. BA‐1000; Vector Lab) using the immunohistochemical procedure as described (Wiley et al., 2016). A negative control without addition of the primary antibodies was also performed. Four mice from each of the three groups, young, old treated with vehicle and old treated with GCV, were analysed; multiple lumbar sagittal sections from each mouse were quantified for nuclear HMGB1 staining in the AF and NP tissue.

### 4.7 mRNA analysis

Total RNA was isolated using TRIzol™ Reagent as per the manufacturer's instructions (Cat. No. 15596026; Thermo Fisher). Real‐time quantitative RT–PCR was run using iTaq™ Universal SYBR^®^ Green One‐Step kit (Cat. No. 1725151; Bio‐Rad) and Bio‐Rad iCycler IQ5 Detection System. Target gene expression was calculated by the comparative *C_T_* method (ΔΔ*C_T_*) and normalized to the GAPDH mRNA level. PCR primers used in the study are as follows: Cdkn2a (p16) forward: AATCTCCGCGAGGAAAGC; Cdkn2a (p16) reverse: GTCTGCAGCGGACTCCAT; MMP13 forward: TCCCTGCCCCTTCCCTATGGT; MMP13 reverse: CTCGGAGCCTGTCAACTGTGGA; GAPDH forward: GAGGCCGGTGCTGAGTAT; GAPDH reverse: GCGGAGATGATGACCCTTTTGG; Aggrecan forward: GCGAAGCAGTACACATCATAGG; Aggrecan reverse: ATACCCCATCCACACGCCCCG

### 4.8 Statistical analysis

Shapiro–Wilk test was used to test for normality. Student's independent *t* test was used to analyse data that were found to be normal. For two‐variable nonparametric data, the Mann–Whitney test was used. Analysis of variance (ANOVA) with Bonferroni correction for multiple comparison was used in cases of data with multivariables. Statistics were derived using GraphPad Prism from GraphPad Software (San Diego, CA). All graphs show mean values with error bars (*SD* or *SEM*, as defined in the figure legends), unless specified otherwise. *p < *0.05 was considered to be significant.

## CONFLICT OF INTEREST

None declared.

## DISCLOSURES

Judith Campisi and Daohong Zhou are co‐founders and advisors of Unity Biotechnology, which develops senolytic drugs.
