# Supplementary material for: Systemic clearance of p16INK4a‐positive senescent cells mitigates age‐associated intervertebral disc degeneration
Source: Aging Cell. 2019 Mar 21;18(3):e12927. doi: 10.1111/acel.12927 (PMC6516165; doi:10.1111/acel.12927)

Supplemental figure S1

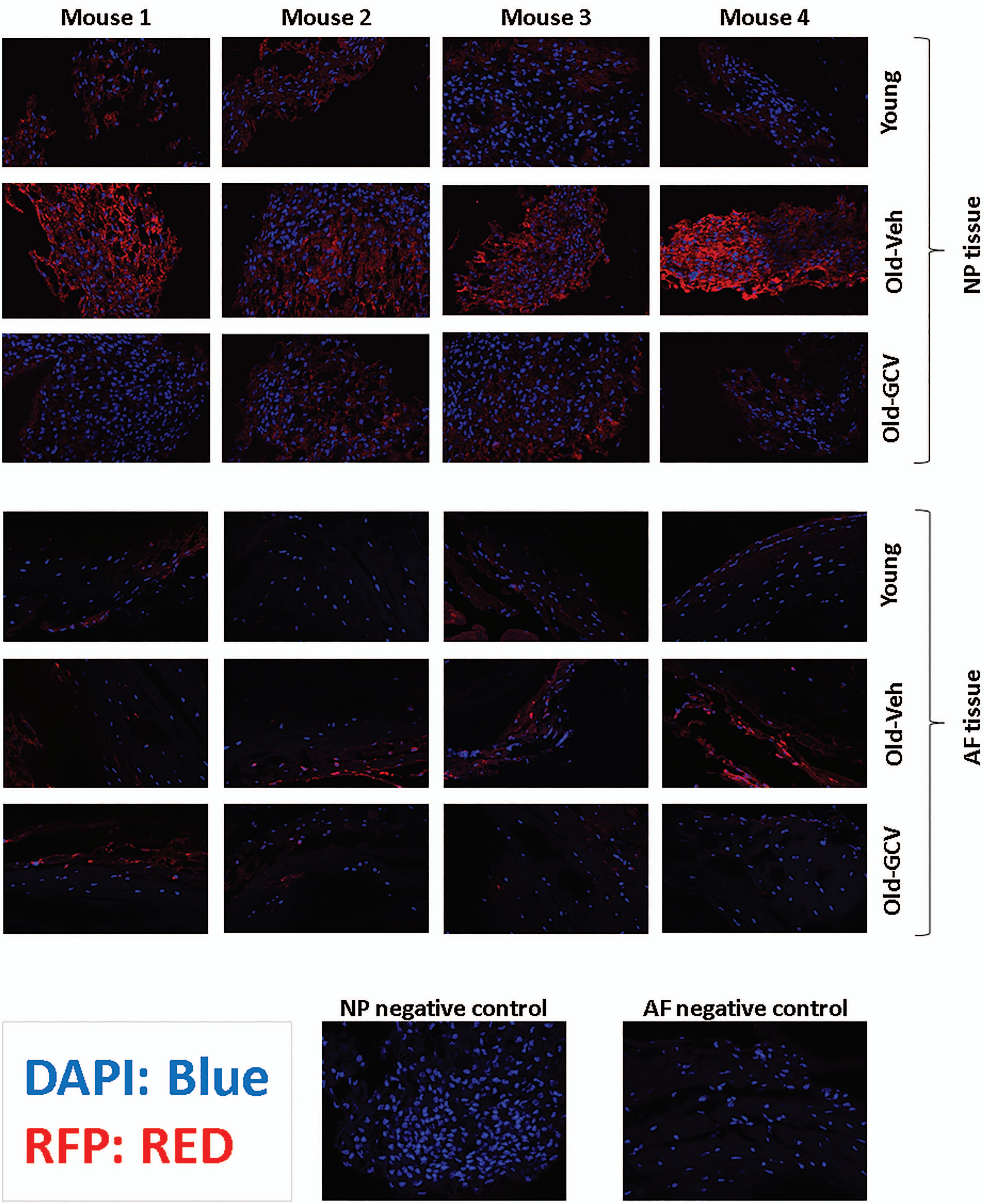

Supplemental figure S2

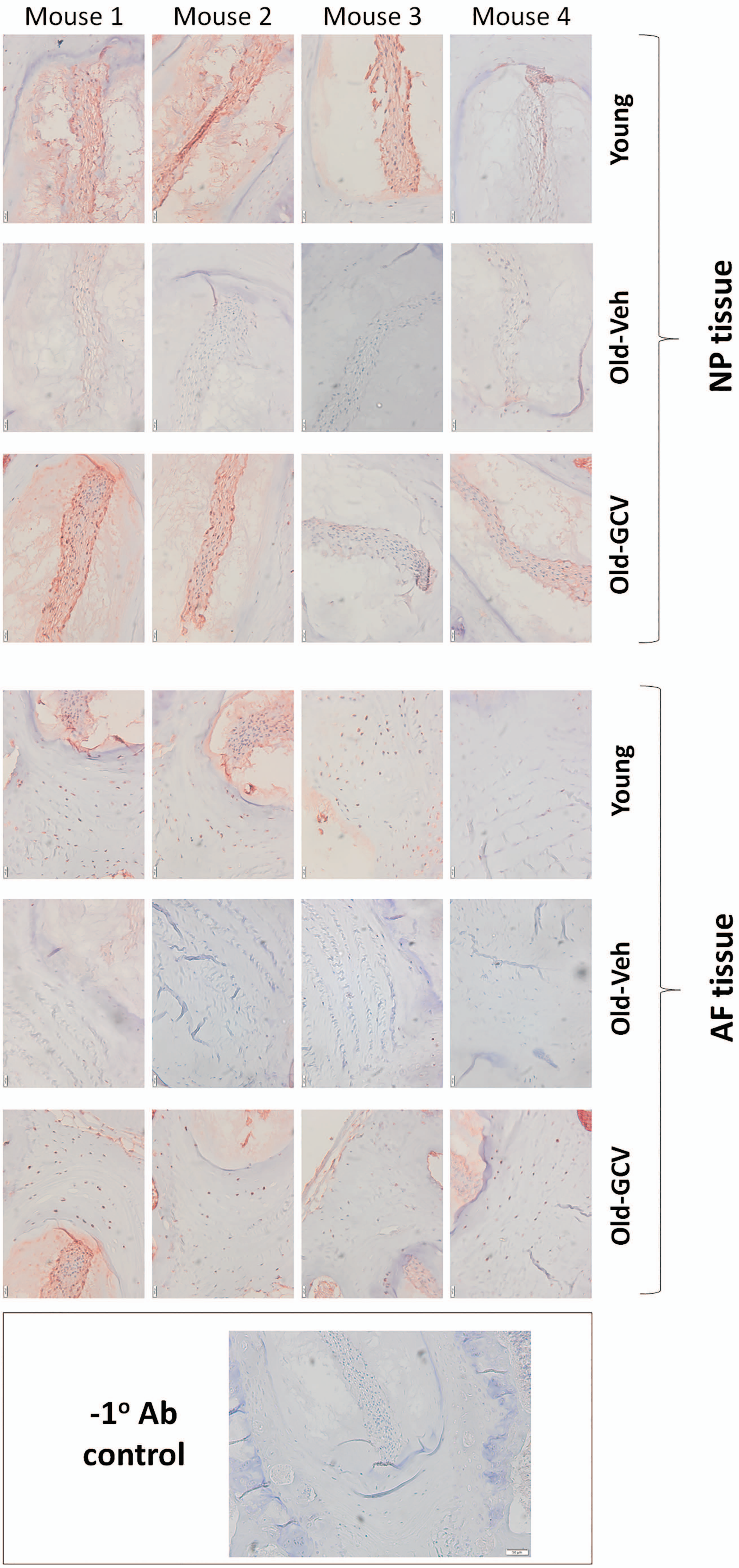

Supplemental figure S3

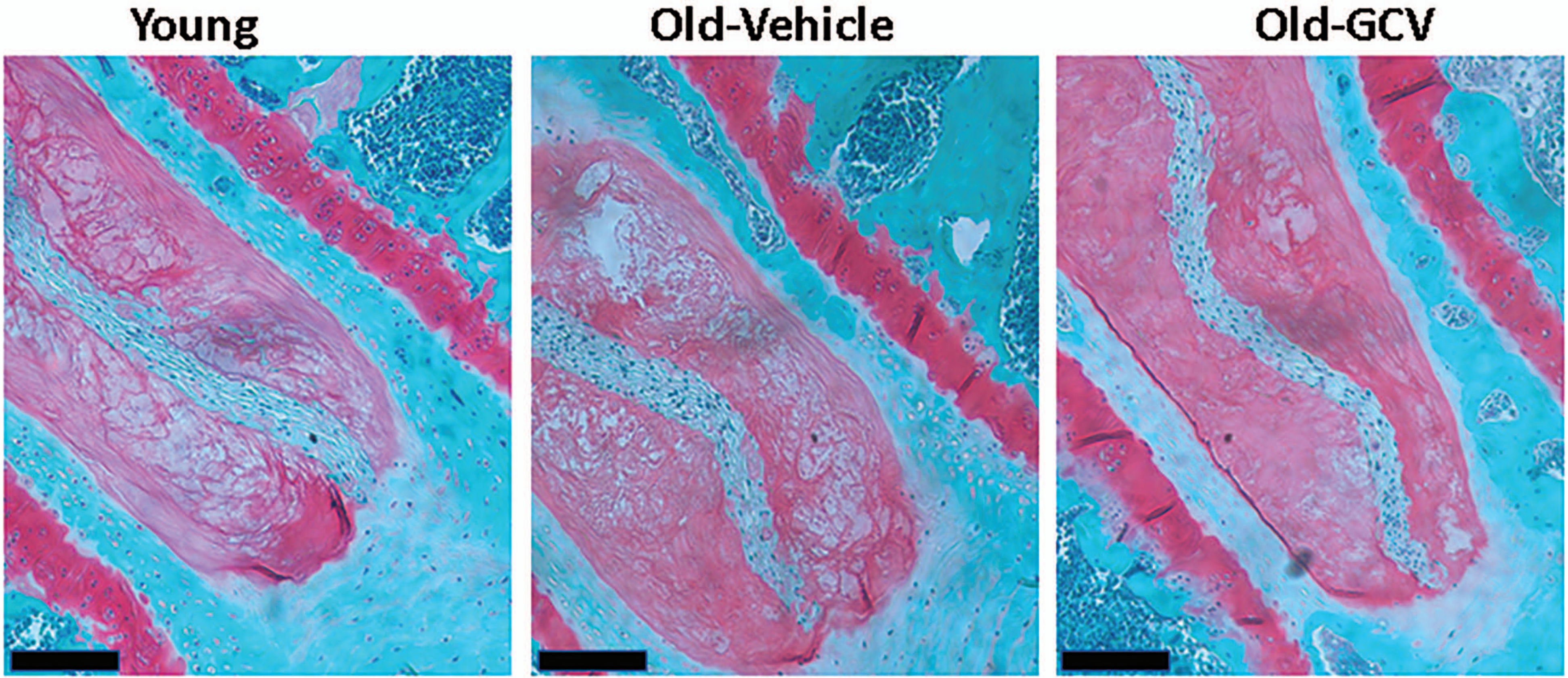

Supplemental figure S4

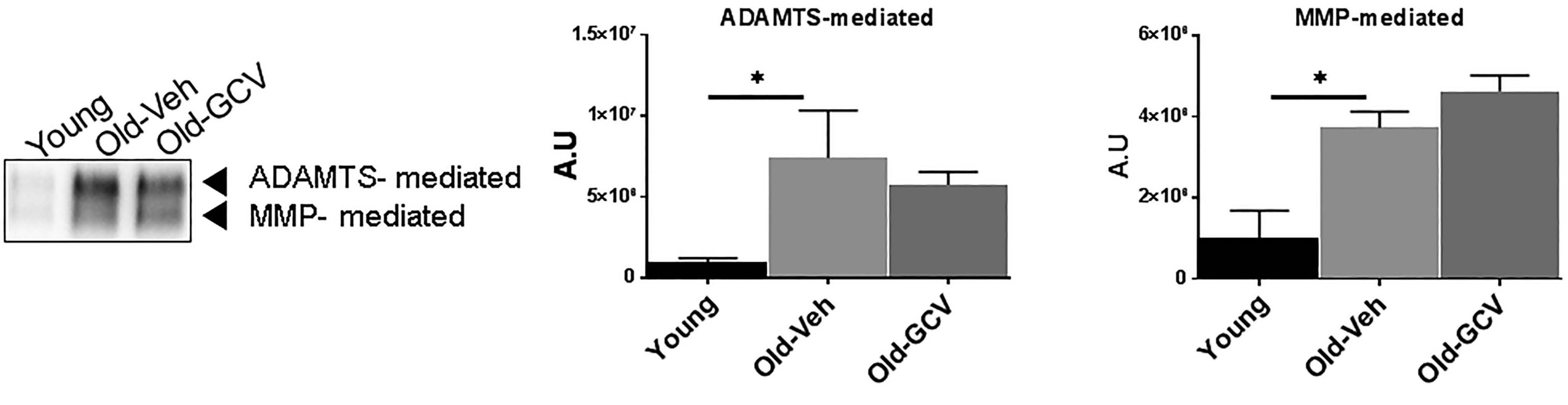

Supplemental figure S5

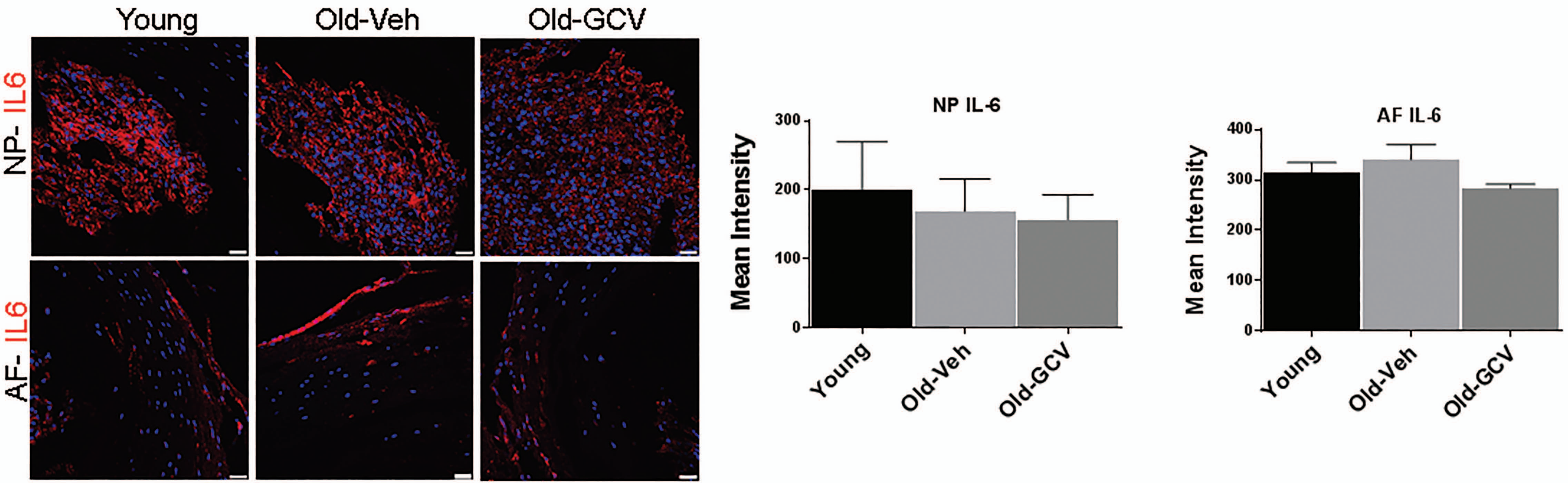

Supplement: Supplementary file 1 [file ACEL-18-e12927-s001.pdf]
